# Infection of Ruminants, Including Pregnant Cattle, with Bungowannah Virus

**DOI:** 10.3390/v12060690

**Published:** 2020-06-26

**Authors:** Andrew J. Read, Deborah S. Finlaison, Peter D. Kirkland

**Affiliations:** Virology Laboratory, Elizabeth Macarthur Agriculture Institute, Woodbridge Road, Menangle, New South Wales 2568, Australia; andrew.j.read@dpi.nsw.gov.au (A.J.R.); deborah.finlaison@dpi.nsw.gov.au (D.S.F.)

**Keywords:** Bungowannah virus, pestivirus F, ruminant infection

## Abstract

Bungowannah virus is a pestivirus known to cause reproductive losses in pigs. The virus has not been found in other species, nor is it known if it has the capacity to cause disease in other animals. Eight sheep, eight calves and seven pregnant cows were experimentally infected with Bungowannah virus. It was found that sheep and calves could be infected. Furthermore, it was shown that the virus is able to cross the bovine placenta and cause infection of the foetus. These findings demonstrate the potential for species other than pigs to become infected with Bungowannah virus and the need to prevent them from becoming infected.

## 1. Introduction

In June 2003 a syndrome of sudden death in sucker pigs, followed by a marked increase in stillborn foetuses and pre-weaning losses, occurred on a large farm in NSW, Australia [1]. Myocarditis and myonecrosis were also observed in affected pigs. A novel pestivirus, known as Bungowannah virus, was subsequently identified [2]. A series of field and laboratory studies have provided strong evidence that Bungowannah virus is the aetiological agent in this disease [2,3,4,5,6,7,8]. Bungowannah virus contains all of the genomic and structural elements of classically described pestiviruses, yet phylogenetic analysis demonstrates that it is genetically remote from any of the other pestivirus species [9,10]. Bungowannah virus is the only extant isolate of *pestivirus F* species [10].

Pestiviruses were initially classified according to their host specificity. Whilst this classification was originally appropriate for classical swine fever virus (CSFV), it was soon shown that bovine viral diarrhea virus (BVDV) and border disease virus (BDV) could naturally infect a variety of ruminants, pigs and other mammals. Recently, CSFV has also been shown to naturally infect cattle [11]. In contrast, Bungowannah virus has only ever been detected in pigs. The origin of this virus is not known, nor what threat it may pose to other species. Bungowannah virus has been shown to replicate in ovine and bovine cells in vitro [12] and so the possibility that it may infect ruminants has been raised. This paper documents the outcome of experimental infections of sheep and cattle with Bungowannah virus. Patterns of virus shedding and pathology are described.

## 2. Materials and Methods 

A series of inoculation experiments were conducted in both sheep and cattle. Cattle were either directly inoculated using intranasal instillation or by co-housing with pigs that were chronically infected with Bungowannah virus. Sheep were either directly inoculated using intranasal instillation or subcutaneous injection or by co-housing with pigs that were chronically infected with Bungowannah virus. The specific details are as follows:

### 2.1. Virus Amplification

The inoculum used for each of the direct inoculation experiments was derived from pooled pig foetal tissues that were passaged once in PK-15 cells (RIE5–1, Collection of Cell Lines in Veterinary Medicine, Friedrich-Loeffler-Institut, Insel Riems, Germany). The titre of infectious virus was also determined by titration in PK-15 cells using standard methods.

### 2.2. Viral Transport Medium

Swabs were collected into 3 mL of sterile phosphate buffered saline (137 mM NaCl, 8 mM Na_2_HPO_4_, 2.7 mM KCl and 1.5 mM KH_2_PO_4_, pH 7.4) containing 0.5% gelatin (*w*/*v*), 5000 IU penicillin/mL, 95,000 IU streptomycin, 50 µg/mL amphotericin B and 0.1% (*w*/*v*) phenol red (PBGS). 

### 2.3. Bungowannah Virus Real-Time Polymerase Chain Reaction (qRT-PCR)

Bungowannah virus RNA was identified from samples using a real-time, reverse transcription PCR (qRT-PCR). The method has been previously described [3]. The fluorescence threshold was set manually at 0.05 and the background was automatically adjusted. qRT-PCR results were expressed as cycle threshold (Ct) values and classified as negative if no amplification was observed after the 45 cycles. For quantification, a 10-fold dilution series of Bungowannah virus RNA standards ranging from 10^7^ to 10^2^ RNA copies/5 µL [6] was included in the assay and the quantity of Bungowannah virus RNA in a sample was determined from the standard curve.

### 2.4. Bungowannah Virus Neutralisation Test

Antibody titres against Bungowannah virus were measured by virus neutralisation test (VNT). The VNT was performed as described previously [5]. Selected serum samples were tested in the VNT in a two-fold dilution series commencing at 1/4. 

### 2.5. Infection of Sheep 

Sheep used in these trials were obtained from a flock that was free of infection with ruminant pestiviruses and had not been vaccinated against pestiviruses. All sheep were tested for anti-pestivirus antibodies using a bovine viral diarrhea virus agarose gel immunodiffusion assay [13] and were found to be negative. 

#### 2.5.1. Direct Inoculation

Six 3-month-old Merino lambs were infected intranasally with 2 mL of cell culture amplified Bungowannah virus (5.6 log_10_ TCID_50_/mL). Two other sheep were inoculated with the same dose subcutaneously while another two other sheep were held as uninfected controls. The inoculated sheep were held in two 11 m^2^ rooms (four intranasally infected sheep in one room, the remaining four infected sheep in the other room). The two uninfected sheep were held in a similar 11 m^2^ room and were not challenged.

Conjunctival, nasal, oral and rectal swabs, along with serum samples, were collected from all sheep prior to exposure to Bungowannah virus and daily for 14 days. Blood samples were subsequently collected approximately weekly until 6 weeks post-exposure. Clinical signs, including rectal temperatures, were also recorded daily for the first 14 days. The swabs and sera were tested for the presence of Bungowannah virus using real-time PCR (qRT-PCR). Serum samples were tested for the presence of antibodies against Bungowannah virus using a VNT.

#### 2.5.2. Exposure to Chronically Infected Pigs

Four 3-month-old Merino lambs were held in a small room (16 m^2^) with three pigs that were chronically infected with Bungowannah virus. The pigs (08-01, 08-05 and 10-01) had been infected in utero and were shown to be shedding Bungowannah virus (oropharyngeal secretions—6.9, 7.0 and 3.4 log_10_ copies/swab, respectively) 7 days prior to the trial [7]. The sheep and the pigs were co-housed for 48 h. During this time there were two periods of six hours of direct physical contact between the sheep and pigs. During the remainder of the time the pigs were separated from the sheep by a mesh partition which allowed for limited direct contact. The clinical examination and sampling were conducted as described above.

### 2.6. Infection of Calves

Calves used in the following trials were obtained from a herd that was free of infection with ruminant pestiviruses and had not been vaccinated against pestiviruses. All calves were tested for anti-pestivirus antibodies using a bovine viral diarrhea virus agarose gel immunodiffusion assay [13] and were found to be negative.

#### 2.6.1. Direct Inoculation

Eight 10-week-old Holstein–Friesian calves were infected intranasally with 2 mL of cell culture-amplified Bungowannah virus (5.6 log_10_ TCID_50_/mL). Two additional calves were held as uninfected controls and did not receive a challenge. The conditions under which they were held, the clinical examination and sampling were conducted as described above for the sheep.

#### 2.6.2. Exposure to Chronically Infected Pigs

Four 5-week-old Holstein–Friesian calves were held with two pigs (08-01 and 08-05; oropharyngeal secretions 5.5 and 7.1 log_10_ copies/swab respectively, 6 days prior to trial) [7] chronically infected with Bungowannah virus. The conditions under which they were held, the clinical examination and sampling were conducted as described above for the sheep.

### 2.7. Infection of Pregnant Cows

Five pregnant Holstein-Friesian and two Illawarra-Shorthorn cows were chosen for the trial. They were obtained from a herd known to be free of infection with ruminant pestiviruses and had not been vaccinated against pestiviruses. All cows were tested for anti-pestivirus antibodies using a bovine viral diarrhea virus agarose gel immunodiffusion assay [13] and found to be negative. They were infected by intranasal instillation of 2 mL of the cell culture amplified Bungowannah virus (4.5 log_10_ TCID/mL). Cows were between 53 and 65 days of pregnancy at the time of inoculation. Nasal and conjunctival swabs and serum samples were collected daily for 14 days following inoculation. The swabs and sera were tested for the presence of Bungowannah virus using qRT-PCR. Serum samples were then collected monthly until calving and were tested for antibodies against Bungowannah virus using a VNT. Pregnancy was monitored by ultrasound examination on a monthly basis. Cows were induced to calve between 255 and 276 days of pregnancy. Serum samples were collected from each calf after birth and prior to suckling. Conjunctival, nasal, oral and rectal swabs and serum samples were collected from calves every 48–72 h for 14 days. Vaginal swabs were also collected from the cows post-partum. A post-mortem examination of the calves was performed between 2 and 4 weeks of age. A wide range of tissues including brain, myocardium and skin (and testicle from a male) were tested for the presence of Bungowannah virus by qRT-PCR. Samples for qRT-PCR were collected by firmly rubbing a swab across the freshly cut surface of a section of the tissue and placed into 3 mL of PBGS. Skin biopsies were stored in 3 mL PBGS. All samples were stored at 4 °C until tested by qRT-PCR and virus isolation. 

### 2.8. Animal Ethics Approval 

The trials described in this paper were approved by the Elizabeth Macarthur Agricultural Institute Animal Ethics Committee. The specific approvals were AEC M09/17 “Studies of the biology of Bungowannah virus infections (PMC) in sheep and cattle” (7 December 2009) and AEC M10/16 “Effects of Bungowannah virus infection in pregnant cattle” (22 December 2010).

## 3. Results

### 3.1. Infection of Sheep

#### 3.1.1. Direct Inoculation

Bungowannah virus RNA was detected between days four and 11 in the nasal swabs of five of the six sheep exposed by intranasal instillation. Ct values ranged between 27.9 and 39.9 (Table 1). Low levels of Bungowannah virus RNA were sporadically detected between days three and 13 of infection in serum samples from four of the six intranasally inoculated sheep. Ct values ranged between 36.5 and 38.1. Two of these sheep had Bungowannah virus RNA detected in serum on three occasions (Table 1). 

Bungowannah viral RNA was not detected in oral, conjunctival or rectal swabs of any of the inoculated or control sheep, nor in serum or nasal swabs for the control sheep or those inoculated subcutaneously. None of the infected sheep developed clinical signs during the course of the infection. The rectal temperatures remained within normal limits. All eight sheep directly inoculated (either intranasally or subcutaneously) developed Bungowannah virus-specific antibodies (Table 2). 

#### 3.1.2. Exposure to Chronically Infected Pigs

Viral RNA was detected only in the nasal swabs of three of the four sheep during the period of co-mingling with the chronically infected pigs. The Ct values ranged from 36.1 to 36.4. Bungowannah virus RNA was not detected in serum or any other swabs during this time and was not detected in serum or any swabs after the pigs were removed from the room. None of the sheep developed Bungowannah virus-specific antibodies.

### 3.2. Infection of Calves

#### 3.2.1. Direct Inoculation

Bungowannah virus RNA was detected in nasal swabs of all eight calves on at least three occasions between days two and 11 (Table 3). Viral RNA was detected sporadically in serum samples from six of these eight calves between days five and 10 of infection and intermittently in the oral swabs from four calves between days four and eight. Bungowannah virus was not detected in any rectal swabs. All eight directly inoculated calves developed antibodies directed against Bungowannah virus by 14 days post-inoculation (Table 4). A very mild nasal discharge was observed in five of the challenged calves. The rectal temperatures remained within normal limits.

#### 3.2.2. Exposure to Chronically Infected Pigs

Viral RNA was detected in the nasal swabs of two of the four calves during the 48 h that they were housed in contact with chronically infected pigs. The virus was detected in the oral swab of another calf on day two and the nasal swab of this same calf on day eight. This calf was the only one of the four to develop antibodies against Bungowannah virus. Neutralising antibodies were first detected 16 days post-exposure. The antibody titre in this calf peaked at 2048 between 63 and 84 days post-inoculation. Bungowannah viral RNA was not detected in any other samples collected. Calves remained healthy throughout the study.

### 3.3. Infection of Pregnant Cows

Each of the seven cows inoculated with Bungowannah virus were found to shed Bungowannah virus RNA in nasal secretions. Shedding began in most cows at day three, with peak virus shedding between days seven and 10. One cow was still shedding viral RNA in nasal secretions on day 14 (Table 5). Bungowannah virus RNA was not detected in conjunctival swabs but was detected in the serum of four of the seven cows for a period of one to three days. No signs of respiratory disease or pyrexia were observed in the cows.

All cows developed neutralising antibodies against Bungowannah virus (Table 6). One cow had developed antibodies by day 11, and the remainder by day 14. Titres peaked on day 44 post infection. Neutralising antibody was detected for the duration of the pregnancy for all cows. A biphasic antibody titre developed in Cow 3 with a peak of 2048 on days 37 and 44, a decline to 512 on days 66 and 100, and then a rise to 1024 on day 128 and 2048 on day 163. 

Pregnancies in the cows were unremarkable with no abnormalities were detected by ultrasound. At birth and before suckling three of the seven calves were found to have antibodies against Bungowannah virus. Bungowannah virus RNA was also detected in ear skin biopsy samples from these same three calves, with Ct values of 26.0, 32.7 and 33.6. The calf with the highest RNA concentration (lowest Ct value) was the progeny of Cow 3. Bungowannah virus RNA was also detected in the serum of this calf. Viral RNA was not detected in conjunctival, oral, nasal or rectal swabs. All calves appeared normal at birth and displayed normal behaviour. The virus was detected in the vaginal swabs from dams of the three positive calves, but not from the other cows. Cow 3 had a Ct value of 30.4, while the other two were 35.8 and 36.6.

Post-mortem examination of all calves was unremarkable, with no gross abnormalities detected. The three calves found to have Bungowannah virus RNA in the skin at birth were euthanased and necropsied at four weeks of age. Viral RNA was again detected in the skin of these calves, and also detected in the testicle of the single male calf among these three (Table 7). Viral RNA was not detected in any other tissues (oesophagus, stomach, duodenum, jejunum, ileum, caecum, colon, liver, bile, mesenteric lymph node, tracheobronchial lymph node, prescapular lymph node, tonsil, thymus, spleen, trachea, lung, thyroid, adrenal gland, kidney, urine, epididymis, ovary, uterus, skeletal muscle, bone marrow, myocardium, cortex, cerebellum or medulla). Virus isolation was attempted on all qRT-PCR positive samples but was not successful.

## 4. Discussion

In this study we have demonstrated that Bungowannah virus can infect a proportion of both cattle and sheep after intranasal or subcutaneous inoculation, although infection was less efficient by the intra-nasal route when compared to pigs [5]. Additionally, viral RNA was detected sporadically at low levels in the serum of a proportion of infected sheep and cattle. These results suggest that following intra-nasal or subcutaneous exposure, the infection is predominantly localized. Systemic infection, as determined by the detection of viraemia, only occurred in a proportion of infections. 

No significant disease was observed in the infected sheep and cattle, despite all the intranasally infected cattle shedding Bungowannah virus RNA in nasal swabs for up to two weeks after infection. The qRT-PCR results indicated that only low levels of virus were shed. The pattern of detection of RNA was consistent with virus replication rather than residual inoculum as there was a short period after inoculation when no RNA was detected. Similarly, viral RNA was detected in the nasal swabs of five of the six sheep infected intranasally, yet no disease was observed. It is interesting to note that the development of the humoral immune response in the two subcutaneously inoculated sheep occurred over a similar period as the intranasally inoculated sheep. We speculate the infection was localised at the injection site in these two animals and, in the absence of a viraemia, shedding via the upper respiratory tract did not occur. The low level of viral RNA detection in nasal secretions of sheep and cattle would suggest that ruminants are unlikely to play a significant role in transmission of the virus.

It was also possible to infect a calf through co-habitation with a chronically infected pig for 48 h. Interestingly, this calf was the most curious of the four calves in the trial and interacted the most with the pigs. It is possible that if given longer exposure to the infected pigs the other calves may have become infected. 

Although all cows became infected, transplacental infection of the calf only occurred in less than half of the cases. Viraemia was associated with in utero infection in two of the four cows in which it was detected. It is noteworthy that Cow 3 developed a rising anti-Bungowannah virus antibody titre during the last trimester of pregnancy. The progeny of this cow was the calf with the highest levels of Bungowannah RNA at birth. We speculate that the dam’s immune system may have been stimulated by transplacental transfer of Bungowannah virus antigen during the last trimester of pregnancy.

Each of the three calves that became infected had seroconverted by the time of birth. Persistent infections were not established, despite the cow being infected at a stage of gestation where immunotolerance and persistent infections would be expected, as usually occurs with BVDV infection. These three calves had significant levels of Bungowannah virus RNA present in skin samples at birth, over 210 days after their dams were infected. We hypothesise that these calves did develop a generalised infection in utero, but subsequently cleared the infection some time prior to birth. The skin and testes were the only sites where the viral RNA was not cleared. The testes are an immunoprivileged site, and so it may be that the infection continued at this site for a longer period of time, perhaps much closer to birth. It has also been suggested that skin is also immunoprivileged [14]. It has been shown that a live BVDV vaccine is able to cross the bovine placenta, cause a prolonged transient infection in the foetus and result in detection of viral RNA in the skin for many weeks after birth [15]. It appears a similar mechanism of infection has occurred in these calves with prolonged detection in the skin. The detection of viral RNA in the post-partum vaginal swabs of the three dams provides further evidence for a prolonged infection in these calves and is comparable to what was observed following infection in pregnant pigs [9].

The timing of infection of pregnant cows was designed to maximise the likelihood of producing persistently infected calves. Infection of cattle with BVDV during the first 90 days of gestation will generally produce calves that are immunotolerant to BVDV [16,17]. The results of this trial indicate that Bungowannah virus and BVDV infections in cattle behave differently, with Bungowannah failing to establish immunotolerance and persistence in the bovine foetus.

Understanding why Bungowannah virus resulted in a humoral response in these calves may lead to a deeper understanding of the functional differences between specific proteins in BVDV and Bungowannah virus and how they do or do not affect evasion of the host innate immune response in the bovine or porcine foetus [18]. 

In conclusion, while cattle and sheep can be infected with Bungowannah virus, they appear to be less susceptible to infection compared to pigs when challenged with a similar dose of virus. When compared to the infection of pigs [9], ruminant species appear to shed less virus and for a shorter period of time. As a result, transmission of Bungowannah virus is likely to be inefficient in ruminants and, without further host adaptation, it will probably not be able to be sustained in these species.

## Figures and Tables

**Table 1:** Bungowannah virus qRT-PCR results from sheep intranasally and subcutaneously inoculated.

		Days Post Inoculation														
Sheep	Sample	0	1	2	3	4	5	6	7	8	9	10	11	12	13	14
**1N**	Nasal	-	-	-	-	36.8	-	-	33.3	-	37.6	34.3	34.1	-	-	-
	Serum	-	-	-	-	-	-	37.1	-	37.8	-	-	36.5	-	-	-
**2N**	Nasal	-	-	-	-	36.0	36.6	32.6	-	34.5	32.2	37.4	37.5	-	-	-
	Serum	-	-	-	-	-	-	-	-	37.7	-	-	-	-	-	-
**3N**	Nasal	-	-	-	-	30.0	32.3	27.9	29.1	29.6	34.2	39.9	-	-	-	-
	Serum	-	-	-	37.2	-	-	-	-	-	-	-	-	-	-	-
**4N**	Nasal	-	-	-	-	29.4	-	34.2	31.1	-	30.5	-	38.4	-	-	-
	Serum	-	-	-	-	-	-	-	38.1	-	36.6	-	-	-	37.1	-
**5N**	Nasal	-	-	-	-	-	33.2	-	-	-	-	-	-	-	-	-
	Serum	-	-	-	-	-	-	-	-	-	-	-	-	-	-	-
**6N**	Nasal	-	-	-	-	-	-	-	-	-	-	-	-	-	-	-
	Serum	-	-	-	-	-	-	-	-	-	-	-	-	-	-	-
**7S**	Nasal	-	-	-	-	-	-	-	-	-	-	-	-	-	-	-
	Serum	-	-	-	-	-	-	-	-	-	-	-	-	-	-	-
**8S**	Nasal	-	-	-	-	-	-	-	-	-	-	-	-	-	-	-
	Serum	-	-	-	-	-	-	-	-	-	-	-	-	-	-	-
**9C**	Nasal	-	-	-	-	-	-	-	-	-	-	-	-	-	-	-
	Serum	-	-	-	-	-	-	-	-	-	-	-	-	-	-	-
**10C**	Nasal	-	-	-	-	-	-	-	-	-	-	-	-	-	-	-
	Serum	-	-	-	-	-	-	-	-	-	-	-	-	-	-	-

A dash indicates that Bungowannah virus RNA was not detected.

**Table 2:** Bungowannah VN titres from sheep intranasally and subcutaneously inoculated.

		Days Post Inoculation						
Sheep	Route of Infection	0	9	15	21	29	36	43
**1N**	Intranasal	-	-	256	128	256	256	256
**2N**	Intranasal	-	-	-	32	32	32	64
**3N**	Intranasal	-	-	64	64	128	128	128
**4N**	Intranasal	-	-	-	64	128	64	256
**5N**	Intranasal	-	-	-	64	256	512	256
**6N**	Intranasal	-	-	-	32	128	128	64
**7S**	Subcutaneous	-	-	-	128	256	256	256
**8S**	Subcutaneous	-	-	64	256	256	256	256
**9C**	Uninfected	-	-	-	-	-	-	-
**10C**	Uninfected	-	-	-	-	-	-	-

A dash indicates that neutralising anti-Bungowannah virus antibodies were not detected.

**Table 3:** Bungowannah virus qRT-PCR results from calves intranasally inoculated.

		Days Post Inoculation														
Calf	Sample	0	1	2	3	4	5	6	7	8	9	10	11	12	13	14
**1N**	Nasal	-	-	-	-	-	-	27.5	34.5	32.5	-	-	-	-	-	-
	Oral	-	-	-	-	-	-	37.6	37.7	-	-	-	-	-	-	-
	Serum	-	-	-	-	-	-	-	-	-	-	-	-	-	-	-
**2N**	Nasal	-	-	34.5	30.4	31.8	31.5	28.1	28.4	-	-	-	-	-	-	-
	Oral	-	-	-	-	-	-	-	-	-	-	-	-	-	-	-
	Serum	-	-	-	-	-	-	-	35.9	-	-	-	-	-	-	-
**3N**	Nasal	-	-	-	-	-	33.9	30.5	31.2	-	37.9	-	-	-	-	-
	Oral	-	-	-	-	-	-	-	-	-	-	-	-	-	-	-
	Serum	-	-	-	-	-	-	-	37.7	-	-	-	-	-	-	-
**4N**	Nasal	-	-	37.0	32.1	-	-	33.0	30.0	32.8	31.2	-	-	-	-	-
	Oral	-	-	-	-	-	-	-	-	-	-	-	-	-	-	-
	Serum	-	-	-	-	-	35.7	-	-	37.5	35.7	-	-	-	-	-
**5N**	Nasal	-	-	33.9	30.2	31.4	27.6	27.1	28.4	29.3	35.2	-	-	-	-	-
	Oral	-	-	-	-	-	-	36.7	-	-	-	-	-	-	-	-
	Serum	-	-	-	-	-	-	-	-	-	-	-	-	-	-	-
**6N**	Nasal	-	-	36.2	34.1	-	31.0	29.7	30.7	27.6	33.6	-	37.4	-	-	-
	Oral	-	-	-	-	-	-	-	-	-	-	-	-	-	-	-
	Serum	-	-	-	-	-	36.3	-	36.3	36.0	37.6	-	-	-	-	-
**7N**	Nasal	-	-		33.1	31.4	32.5	30.1	29.2	29.7	-	-	-	-	-	-
	Oral	-	-	-	-	36.6		36.6	-	-	-	-	-	-	-	-
	Serum	-	-	-	-	-	-	37.3	36.4		36.5	37.7	-	-	-	-
**8N**	Nasal	-	-	-	-	31.7	31.6	30.3	31.4	33.4	-	-	-	-	-	-
	Oral	-	-	-	-	-	-	-	-	37.5	-	-	-	-	-	-
	Serum	-	-	-	-	-	37.3	-	-	-	-	-	-	-	-	-
**9C**	Nasal	-	-	-	-	-	-	-	-	-	-	-	-	-	-	-
	Oral	-	-	-	-	-	-	-	-	-	-	-	-	-	-	-
	Serum	-	-	-	-	-	-	-	-	-	-	-	-	-	-	-
**10C**	Nasal	-	-	-	-	-	-	-	-	-	-	-	-	-	-	-
	Oral	-	-	-	-	-	-	-	-	-	-	-	-	-	-	-
	Serum	-	-	-	-	-	-	-	-	-	-	-	-	-	-	-

A dash indicates that Bungowannah virus RNA was not detected. Calves 9C and 10C were uninfected.

**Table 4:** Bungowannah VN titres from calves intranasally inoculated.

		Days Post Inoculation							
Calf	Route of Infection	0	7	14	21	28	35	42	49
**1N**	Intranasal	-	-	16	512	1024	2048	2048	4096
**2N**	Intranasal	-	-	64	256	1024	2048	2048	2048
**3N**	Intranasal	-	-	32	256	1024	4096	8192	4096
**4N**	Intranasal	-	-	64	2048	2048	16,384	8192	2048
**5N**	Intranasal	-	-	32	128	512	2048	1024	4096
**6N**	Intranasal	-	-	16	1024	256	2048	8192	4096
**7N**	Intranasal	-	-	128	1024	4096	16,384	32,768	32,768
**8N**	Intranasal	-	-	16	128	1024	2048	4096	8192
**9C**	Uninfected	-	-	-	-	-	-	-	-
**10C**	Uninfected	-	-	-	-	-	-	-	-

A dash indicates that neutralising anti-Bungowannah virus antibodies were not detected. Calves 9C and 10C were uninfected.

**Table 5:** qRT-PCR results for pregnant cows infected with Bungowannah virus.

		Days Post Inoculation														
Cow		0	1	2	3	4	5	6	7	8	9	10	11	12	13	14
**1**	Nasal	-	-	-	36.6	-	31.8	33.9	33.6	30.2	31.0	31.6	32.7	-	-	
	Serum	-	-	-	-	-	-	-	-	-	33.4	35.5	35.0	-	-	-
**2**	Nasal	-	-	-	30.5	35.6	30.9	36.2	28.7	29.3	27.8	31.8	34.0	34.3	-	-
	Serum	-	-	-	-	-	-	-	-	-	-	-	-	-	-	-
**3**	Nasal	-	-	-	35.6	36.0	-	37.4	35.4	33.2	33.6	31.8	34.6	35.4	-	-
	Serum	-	-	-	-	-	-	-	-	36.8	-	-	-	-	-	-
**4**	Nasal	-	-	-	-	34.3	34.0	29.8	28.5	28.5	33.1	-	37.4	-	-	-
	Serum	-	-	-	-	-	-	37.9	27.2	34.0	37.5	-	-	-	-	-
**5**	Nasal	-	-	-	-	-	-	37.4	36.3	-	-	-	37.8	36.4	34.7	32.2
	Serum	-	-	-	-	-	-	-	-	-	-	-	-	-	-	-
**6**	Nasal	-	-	-	37.3	31.8	36.7	35.8	31.1	32.1	-	-	-	-	-	-
	Serum	-	-	-	-	-	-	-	-	-	-	-	-	-	-	36.5
**7**	Nasal	-	37.4	35.4	36.2	-	33.4	32.2	29.7	30.5	27.2	31.8	35.6	-	-	-
	Serum	-	-	-	-	-	-	-	-	-	-	-	-	-	-	-

A dash indicates that Bungowannah virus RNA was not detected.

**Table 6:** Bungowannah VN titres from pregnant cows intranasally inoculated.

	Days Post Inoculation												
Cow	0	7	11	14	21	31	37	44	52	66	100	128	163
**1**	-	-	-	32	1024	2048	4096	4096	2048	1024	1024	512	1024
**2**	-	-	-	32	128	512	512	1024	256	512	512	256	128
**3**	-	-	-	8	256	512	2048	2048	1024	512	512	1024	2048
**4**	-	-	-	8	128	512	1024	2048	512	512	512	512	256
**5**	-	-	8	8	128	512	256	256	128	512	128	256	64
**6**	-	-	-	8	128	512	512	1024	512	NT	2048	512	1024
**7**	-	-	-	16	16	64	64	64	64	64	64	64	32

A dash indicates that neutralising anti-Bungowannah virus antibodies were not detected.

**Table 7:** Summary of laboratory results for progeny of cows infected with Bungowannah virus.

Calf	Gestational Age at Infection (Days)	Gestational Age at Birth (Days)	Skin (Copies/mL)	Serum (Copies/mL)	Testicle (Copies/mL)	VNT
**1**	63	274	1.5 × 10^6^	-	Female	>512
**2**	65	276	8.1 × 10^4^	-	Female	>512
**3**	65	276	2.4 × 10^7^	5.0 × 10^2^	6.4 × 10^5^	>512
**4**	65	274	-	-	-	-
**5**	65	276	-	-	-	-
**6**	63	274	-	-	-	-
**7**	53	255	-	-	Female	-

A dash indicates that a result was undetected. Calf numbers correspond to cow numbers.
